# Direct pancreatic duct orifice incision for complete obstruction after intraductal radiofrequency

**DOI:** 10.1055/a-2846-4828

**Published:** 2026-05-05

**Authors:** Shintaro Kawasaki, Takanori Kanai, Motohiko Kato

**Affiliations:** 1 Center for Diagnostic and Therapeutic EndoscopyKeio University School of MedicineTokyoJapan; 2 Division of Gastroenterology and Hepatology, Department of Internal MedicineKeio University School of MedicineTokyoJapan

**Fig. 1:**
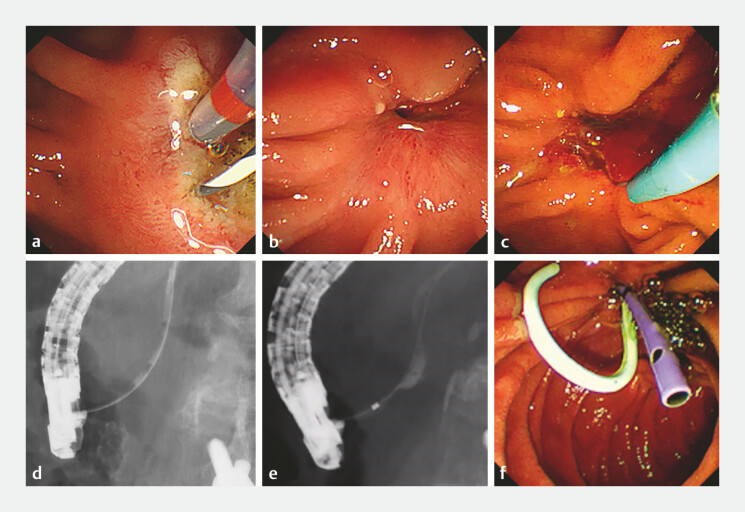
**a** During ID-RFA, guidewires were placed in both the bile duct and the pancreatic duct, allowing estimation of the relative positions of the biliary and pancreatic duct orifices. **b** The biliary orifice could be clearly identified, but the pancreatic duct orifice was not identified. **c** Referring to the previous ERCP images and confirming that the pancreatic orifice was located slightly below the biliary orifice, a precut was performed at the pancreatic orifice ( **a** ). **d** and **e** Comparison of pancreatography before and after ID-RFA. During ID-RFA, the pancreatic duct showed no stenosis ( **d** ). The pancreatic duct in the pancreatic head showed severe stricture, with marked dilation of the distal pancreatic duct ( **e** ). **f** Biliary and pancreatic duct stents were successfully placed. ERCP, endoscopic retrograde cholangiopancreatography; ID-RFA, intraductal radiofrequency ablation.

Intraductal radiofrequency ablation (ID-RFA) has been reported as an effective treatment for residual or recurrent intraductal lesions after endoscopic papillectomy 1 2 . However, ID-RFA targeting the papillary legion may cause delayed bile and pancreatic duct strictures 3 . We report a case in which pancreatic duct stenting was successfully achieved using direct pancreatic orifice incision with a precut knife for ID-RFA-induced benign strictures. A 76-year-old woman with a history of endoscopic submucosal dissection including papilla developed recurrent papillary adenoma with intraductal extension into the bile and pancreatic ducts. She underwent snare polypectomy, APC, and ID-RFA, followed by the placement of the biliary metallic stent and the pancreatic plastic stent ( Fig. 1 **a** ). After the removal of stents on postoperative day (POD) 27, the patient, who had upper abdominal pain, an amylase level of 1,377 U/l, and dilation of the pancreatic duct, was diagnosed with obstructive pancreatitis on POD 45. Though transpapillary pancreatic duct drainage was attempted, the pancreatic orifice could not be identified, and even guidewire cannulation was unsuccessful ( Fig. 1 **b** ). Therefore, based on previous cannulation images indicating that the pancreatic orifice was inferior to the biliary orifice, a mucosal incision was made at the presumed pancreatic orifice using a precut knife ( Fig. 1 **c** and Video 1 ; Needle Cut 3V, Olympus, Tokyo, Japan). Successful guidewire insertion into the pancreatic duct was eventually achieved. As a catheter could not be inserted into the pancreatic duct stricture, balloon dilation was performed using a tapered-tip 4mm balloon (REN, Kaneka Medics, Tokyo, Japan; Fig. 1 **d** and Fig. 1 **e** ). A 4-Fr pancreatic duct stent was successfully placed ( Fig. 1 **f** ). The patient was discharged 6 days later and has remained well in the 75-day follow-up. With the increasing use of ID-RFA for biliary and pancreatic intraductal lesions, complete pancreatic duct obstruction could be encountered more frequently. In such situations, pancreatic orifice incision using a precut knife could be a useful and less invasive option of rescue technique.

Direct pancreatic duct orifice incision for complete obstruction and successful pancreatic duct cannulation.Video 1

Endoscopy_UCTN_Code_CCL_1AZ_2AF
